# An objective model for diagnosing comorbid cognitive impairment in patients with epilepsy based on the clinical-EEG functional connectivity features

**DOI:** 10.3389/fnins.2022.1060814

**Published:** 2023-01-12

**Authors:** Zhe Ren, Yibo Zhao, Xiong Han, Mengyan Yue, Bin Wang, Zongya Zhao, Bin Wen, Yang Hong, Qi Wang, Yingxing Hong, Ting Zhao, Na Wang, Pan Zhao

**Affiliations:** ^1^Department of Neurology, Zhengzhou University People’s Hospital, Zhengzhou, Henan, China; ^2^Department of Neurology, Henan Provincial People’s Hospital, People’s Hospital of Zhengzhou University, Zhengzhou, Henan, China; ^3^Department of Rehabilitation, The First Hospital of Shanxi Medical University, Taiyuan, Shanxi, China; ^4^School of Medical Engineering, Xinxiang Medical University, Xinxiang, Henan, China; ^5^School of Life Sciences and Technology, Xi’an Jiaotong University, Xi’an, Shaanxi, China; ^6^Department of Neurology, People’s Hospital of Henan University, Zhengzhou, Henan, China

**Keywords:** epilepsy, cognitive impairment, EEG, phase locking value, GBDT, AdaBoost, diagnostic model, Fisher score

## Abstract

**Objective:**

Cognitive impairment (CI) is a common disorder in patients with epilepsy (PWEs). Objective assessment method for diagnosing CI in PWEs would be beneficial in reality. This study proposed to construct a diagnostic model for CI in PWEs using the clinical and the phase locking value (PLV) functional connectivity features of the electroencephalogram (EEG).

**Methods:**

PWEs who met the inclusion and exclusion criteria were divided into a cognitively normal (CON) group (*n* = 55) and a CI group (*n* = 76). The 23 clinical features and 684 PLV*_*EEG*_* features at the time of patient visit were screened and ranked using the Fisher score. Adaptive Boosting (AdaBoost) and Gradient Boosting Decision Tree (GBDT) were used as algorithms to construct diagnostic models of CI in PWEs either with pure clinical features, pure PLV*_*EEG*_* features, or combined clinical and PLV*_*EEG*_* features. The performance of these models was assessed using a five-fold cross-validation method.

**Results:**

GBDT-built model with combined clinical and PLV*_*EEG*_* features performed the best with accuracy, precision, recall, F1-score, and an area under the curve (AUC) of 90.11, 93.40, 89.50, 91.39, and 0.95%. The top 5 features found to influence the model performance based on the Fisher scores were the magnetic resonance imaging (MRI) findings of the head for abnormalities, educational attainment, PLV*_*EEG*_* in the beta (β)-band C3-F4, seizure frequency, and PLV*_*EEG*_* in theta (θ)-band Fp1-Fz. A total of 12 of the top 5% of features exhibited statistically different PLV*_*EEG*_* features, while eight of which were PLV*_*EEG*_* features in the θ band.

**Conclusion:**

The model constructed from the combined clinical and PLV*_*EEG*_* features could effectively identify CI in PWEs and possess the potential as a useful objective evaluation method. The PLV*_*EEG*_* in the θ band could be a potential biomarker for the complementary diagnosis of CI comorbid with epilepsy.

## Introduction

Cognitive impairment (CI) is one of the very common comorbidities occurring in 70–80% of patients with epilepsy (PWEs) (Helmstaedter and Witt, 2017). Previous studies have revealed several factors that may induce CI in PWEs, including age at onset, duration of illness, surgical head trauma, perinatal injury, temporal lobe epilepsy, hippocampal abnormalities, seizures, status epilepticus, medications, and psychiatric factors (Black et al., 2010; Titiz et al., 2014; Vrinda et al., 2019; Jarcuskova et al., 2020; Wang et al., 2020; Novak et al., 2022a). Furthermore, interictal epileptiform discharges (IEDs) in electroencephalogram (EEG) recordings are an important indicator of CI in PWEs (Ung et al., 2017; Gavrilovic et al., 2019; Balcik et al., 2020), but the exact role of EEG in diagnosing CI in such patients has rarely been studied.

Cognitive scales serve as the primary method for diagnosing CI, with the Montreal Cognitive Assessment (MoCA) scale considered the most appropriate and more sensitive than the Mini-Mental State Examination (MMSE) scale for screening cognitive impairment in epileptic individuals (Montano-Lozada et al., 2021; Huang et al., 2022; Novak et al., 2022b). Notably, the MoCA-30 point scale is superior to the MoCA-20 scale for CI assessment in clinical practices (Bergeron et al., 2017; Del Brutto et al., 2019; Rodrigues et al., 2020; Melikyan et al., 2021). However, the scale has some shortcomings, most notably its susceptibility to subjective factors from both patients and physicians, which may lead to errors in the test. Although the MoCA scale is well suited to screening for CI in epileptic patients, however, it is a generic neurological screening tool for cognitive assessments. Therefore, there is an urgent need for developing an efficient objective assessment indicator for cognitive functions, specifically for individuals with epileptic symptoms.

Electroencephalogram plays a vital role in the diagnosis and management of epilepsy, as it provides an objective and accurate response to functional changes in the brain, thus avoiding the influence of subjective factors in the patient. A growing body of research has demonstrated a strong correlation between altered cognitive functions and the neural connectivity of different brain regions (He et al., 2018; Fadaie et al., 2021; Duma et al., 2022). Functional connectivity is a type of neural connectivity that mediates the temporal correlation between neurophysiological events at different brain regions and is primarily used to measure the degree of dependency and correlation between the signals. The phase locking value (PLV) is one of the quantitative indicators for functional connectivity (Elahian et al., 2017; Duma et al., 2021). Furthermore, EEG-based functional connectivity is employed to predict vagus nerve stimulation (VNS) responsiveness in children with refractory epilepsies (Ma et al., 2022), as well as to diagnose CI in patients comorbid with Parkinson’s disease (PD) (Cai et al., 2021). However, this approach has not been applied to the diagnosis of cognitive dysfunctions in PWEs. The Adaptive Boosting (AdaBoost) and Gradient-Boosted Decision Trees (GBDT) are classic algorithms for ensemble learning (EL) and have been widely used in areas of neurologic disorders such as epilepsy, Alzheimer’s disease (AD), PD, etc. (Peng et al., 2020; Wenbo et al., 2021; Zhang S. et al., 2021; Edeh et al., 2022). These follow the models constructed based on the clinical and PLV*_*EEG*_* functional connectivity features of EL algorithms and have shown the potential of an efficient objective evaluation tool for diagnosing CI in PWEs.

Here, we used EL algorithms to construct three distinct models for the diagnosis of CI in PWEs, purely based on the clinical and PLV*_*EEG*_* features. Additionally, we investigated to identify potential biomarkers for the diagnosis of cognitive functions in PWEs.

## Materials and methods

### Selection of the participants

A total of 131 PWEs from the outpatient clinic of the Epilepsy Center of Henan Provincial People’s Hospital between June 2018 and May 2022 were retrospectively screened and enrolled in the study. The inclusion criteria were: (1) the patient must meet the criteria of the International League Against Epilepsy (ILAE) for the diagnosis of epilepsy, seizures, and other epileptic syndromes (Fisher et al., 2014); (2) the age range at the time of consultation must be 12–60 years; (3) the patient must had a MoCA test at the time of consultation and should not have any history of MoCA scale testing in the last year; (4) at least 20 min of outpatient scalp EEG at the time of consultation, along with the availability of retrospective EEG data; and (5) the patient must have a complete clinical history and previous cranial MRI findings. Subjects were excluded if: (1) the patient’s age was less than 12 years or more than 60 years at the time of consultation; (2) the patient was diagnosed with psychogenic non-epileptic seizures, or epilepsy syndrome; (3) the patient was treated with drugs other than antiseizures medications that affect cognitive functions, such as benzodiazepines, anti-psychotics, and memory-enhancing drugs, at the time of consultation; and (4) the patient was missing the 20-min EEG recording data at the time of the enrollment.

**FIGURE 1:**
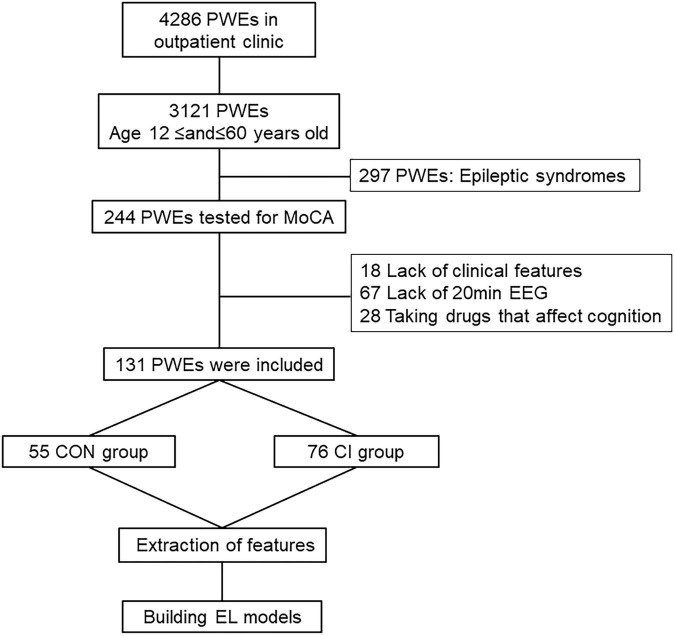
Flow chart. PWEs, patients with epilepsy; MoCA, Montreal Cognitive Assessment; EEG, electroencephalogram; CI, cognitive impairment; CON, cognitively normal; EL, ensemble learning.

Based on the patients’ MoCA scores during their visits to the epilepsy clinic, 131 PWEs were recruited for the study and were subsequently divided into the control (CON) group (MoCA ≥ 26; *n* = 55) and the CI group (MoCA < 26; *n* = 76) (Figure 1 and Table 1). The study was approved by the Ethics Committee of Henan Provincial People’s Hospital and all eligible subjects signed the written informed consent before their final recruitment to the study.

**TABLE 1:** Types of epilepsy in patients with epilepsy used in the study.

Epil. type	Unitemp.	Bitemp.	Par.	Occ.	Central	Front.	Undetermined
CON (*n* = 55)	25	2	0	9	8	2	9
CI (*n* = 76)	44	4	3	7	7	3	8

Epil. type, epilepsy type; Unitemp, unitemporal; Bitemp, bitemporal; Par, parietal; Occ, occipital; Front, frontal.

### Clinical features

Based on the patients’ medical history and clinical investigations at the time of the current visit to the epilepsy clinic, 23 clinical features were identified, in conjunction with previous studies: (1) age; (2) age at the first onset; (3) time from the first onset to current visit (Black et al., 2010); (4) gender; (5) family history of epilepsy (defined as whether a first or second degree relative had epilepsy); (6) history of previous head surgery or trauma; (7) history of previous the central nervous system (CNS) infections; (8) history of perinatal injuries due to premature birth, obstructed labor, hypoxia, and/or intracranial hematoma; (9) TLE; (10) MRI of the head for abnormalities; (11) hippocampal atrophy, or sclerosis (Titiz et al., 2014); (12) different types of seizures like generalized, focal, or both; (13) status epilepticus; (14) generalized tonic-clonic seizures (GTCS); (15) seizure frequency in the last year (Wang et al., 2020) (rare: ≤1 event; occasional: 2–3 events; frequent: ≥4 events); (16) class of antiseizures medications (Wang et al., 2020); (17) valproate (VPA) therapy in the last year; (18) phenytoin (PHT) therapy in the last year; (19) topiramate (TPM) therapy in the last year; (20) aura of epilepsy; (21) anxiety [according to the Hamilton Anxiety Inventory (HAI) scale rating: none, possible, definitely, or definitely obvious]; (22) depression [according to the Hamilton Depression Inventory (HDI) scale rating: none, possible, or definite]; and (23) educational attainment (≤6 years, 7–9 years, 10–12 years, or ≥13 years) (Table 2).

**TABLE 2:** Demographic information and clinical characteristics.

Clinical features	CON group (*n* = 55)	CI group (*n* = 76)	*P*-value
Age. y, mean ± SD	26.38 ± 10.49	31.34 ± 13.93	0.061
Age at first onset. y, mean ± SD	18.76 ± 11.02	20.71 ± 14.74	0.788
Time from first onset to current visit. y, mean ± SD	7.44 ± 7.79	10.63 ± 8.14	0.009*
Female	24	39	0.385
Family history of epilepsy. Y, *n*	2	5	0.730
History of previous head surgery or trauma. Y, *n*	6	17	0.089
History of previous CNS infections. Y, *n*	8	18	0.196
History of perinatal injury. Y, *n*	4	8	0.741
TLE. Y, *n*	27	48	0.108
MRI of the head for abnormalities. Y, *n*	28	51	0.061
Hippocampal atrophy, sclerosis. Y, *n*	14	37	0.004*
Seizure type, *n*			0.875
Generalized	13	21	
Focal	7	9	
Both	35	46	
Status epilepticus. Y, *n*	4	15	0.080
GTCS. Y, *n*	45	67	0.309
Seizure frequency, *n*			0.006*
Rare	17	12	
Occasionally	15	11	
Frequent	23	53	
Class of antiepileptic drugs ≥2. Y, *n*	18	41	0.016*
VPA. Y, *n*	17	40	0.013*
PTH. Y, *n*	1	2	1.000
TPM. Y, *n*	3	4	1.000
Aura of epilepsy. Y, *n*	22	24	0.319
Anxiety, *n*			0.444
None	14	12	
Possible	13	21	
Definitely	25	35	
Definitely obvious	3	8	
Depression, *n*			0.555
None	23	25	
Possible	31	48	
Definitely	1	3	
Educational attainment, *n*			<0.001*
≤6 y	1	23	
7–9 y	11	18	
10–12 y	15	19	
≥13 y	28	16	

y, year; Y, yes; CNS, central nervous system; TLE, temporal lobe epilepsy; MRI, magnetic resonance imaging; GTCS, generalized tonic-clonic seizures; VPA, valproate; PHT, phenytoin, TPM, topiramate.

*P* < 0.05 is considered as statistically significant. *The features that have statistically significance. For continuous variables, independent-samples *t*-test or Mann–Whitney *U*-test was carried out. For categorical variables, chi-square test or Fisher’s exact test were carried out.

### EEG acquisition and preprocessing

All patients in both CON and CI groups had scalp EEG recordings monitored for at least 20 min during this visit. All tests were performed in the awake closed-eye state, while EEG recordings performed during the sleep and awake open-eye states were excluded. The EEG-1200°C machine (Nihon Kohden, Tokyo, Japan), with a sampling frequency of 256 Hz, an amplification multiplier of 1000×, a low-pass filter of 70 Hz, and a high-pass filter of 0.5 Hz, was used for this study. This system uniformly used the international 10–20 lead system for placing the scalp electrodes, including 19 recording leads, namely Fp1, Fp2, Fz, Cz, Pz, C3, C4, T3, T4, T5, T6, F3, F4, F7, F8, O1, O2, P3, and P4, and 2 reference leads A1 and A2.

Preprocessing of EEG data was performed using the EEGLAB toolbox in MATLAB software (Mathworks Inc., USA) (Delorme and Makeig, 2004). Briefly, the EEG recordings were first filtered to extract only the 0.5–30 Hz recordings. Afterward, the artifacts of eye movements in electromyogram (EMG) were removed using independent component analysis. Finally, the 20-min EEG recording of each patient was intercepted into 6 s segments, and PLV*_*EEG*_* features were extracted.

### Parameters setting for AdaBoost and GBDT

AdaBoost and GBDT are typical methods of boosting algorithm. In the AdaBoost model, the number and learning rate of base classifiers were also determined by grid search, ranging from 50 to 150 and 0 to 1, respectively and the algorithm of AdaBoost set to SAMME.R. The base classifier of AdaBoost was SVM, the kernel was RBF and the C and gamma of which were also determined by grid search, ranging from 2^–10^ to 2^10^ and 0.0001 to 10, respectively. Other parameters were set to default values. In the GBDT model, the number, learning rate, and subsample of base classifiers were also determined by grid search, ranging from 50 to 150, 0 to 1 and 0.5 to 0.8, respectively. The base classifier of GBDT was CART, the max depth and the max leaf nodes of which were also determined by grid, search ranging from 10 to 15 and 10 to 30, respectively. Other parameters were set to default values. In order to reduce the contingency and improve the generalization ability, the five-fold cross-validation method was used to evaluate the performance of the model and select the best model. All of the above algorithms were programmed and realized by sklearn in PyCharm IDE using Python 3.7. The computer system is windows 10 professional, the CPU is Inter Core i7-10700K Processor @3.9 GHz, and the RAM is 32 GB. The final parameters of the model are shown in Table 3.

**TABLE 3:** The parameters of the models.

AdaBoost	Value	GBDT	Value
**Clinical feature-based model**			
Base_estimator	SVC	Base_estimator	CART
N_estimators	60	N_estimators	90
Learning_rate	0.2	Learning_rate	0.5
C	1024	Subsample	0.8
Gamma	0.0025	Max_depth	8
Kernel	RBF	Max_leaf_nodes	15
**PLV*_*EEG*_* feature-based model**			
base_estimator	SVC	Base_estimator	CART
N_estimators	100	N_estimators	90
Learning_rate	0.1	Learning_rate	0.2
C	256	Subsample	0.7
Gamma	0.25	Max_depth	10
Kernel	RBF	Max_leaf_nodes	13
**Combined clinical-PLV*_*EEG*_* feature-based model**			
Base_estimator	SVC	Base_estimator	CART
N_estimators	80	N_estimators	110
Learning_rate	0.3	Learning_rate	0.3
C	64	Subsample	0.7
Gamma	0.0125	Max_depth	12
Kernel	RBF	Max_leaf_nodes	15

### PLV-based functional connectivity features

Phase locking value is a type of connection characteristic, which quantifies the degree of phase synchronization between the two EEG signals (Aydore et al., 2013; Leguia et al., 2021). The Hilbert transform was first applied to the preprocessed EEG data to calculate the instantaneous amplitude and instantaneous phase for each lead site. The PLV indicator was then calculated using the following formula:


$$PLV_{t}=\frac{1}{N}\left|\sum_{n=1}^{N}exp\left(j\theta (t,n)\right)\right|$$

Where N denoted the number of EEG segments per subject, θ(*t*,*n*) presented the instantaneous phase difference between different leads of the same segment, *exp*(*j*θ(*t*,*n*)) represented the complex signal obtained with the help of Euler’s formula using phase, and $\sum_{n\,1}^{N}e\,x\,p\,\left(j\,\theta \,\left(t,n\right)\right)$ represented the superimposed value of the complex signals of all segments of a patient, which was averaged to obtain the PLV feature value of a subject.

The PLV feature was then quantized into a value in the range [0,1]. When PLV = 1, the phase difference between the two signals was constant, i.e., perfectly synchronized. When PLV = 0, the phase difference was uniformly distributed over the complex plane unit circle according to time, indicating that there was no synchronization. Between 0 and 1, the signal difference exhibited an “overall convergence” nature, such that as PLV tended to 1, two close signals exhibited better synchronization.

**FIGURE 2:**
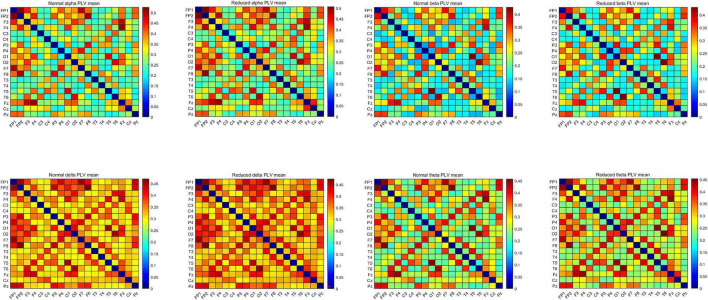
Mean PLV*_*EEG*_* features in the four frequency bands for the CON and the CI groups of PWEs. PWEs, patients with epilepsy; PLV, phase locking value; CI, cognitive impairment; CON, cognitively normal.

Since it would be more accurate to calculate the instantaneous phase of narrowband signals using the Hilbert transform, the preprocessed EEG segments were divided into four narrow bands according to different frequency ranges, namely delta (δ) (1–4 Hz), θ (4–7), alpha (α) (8–13 Hz), and β (14–30 Hz) bands. The PLV*_*EEG*_* values of these four frequency bands were calculated separately for 200 windows (6 s) of each subject’s 20-min EEG recording. Finally, 200 PLV*_*EEG*_* feature matrices of 19 × 19 in each of the four frequency bands were obtained for each subject and averaged into a single matrix for each frequeny band, so that each subject ended up with a total of four feature matrices for four frequency bands. These PLV*_*EEG*_* feature matrices would be further filtered and sorted characterized (Figure 2).

### Feature extraction

As shown earlier, 23 clinical features were selected based on the previous studies and contents of available medical records. The EEG records of all subjects were divided into four different frequency bands. For each subject’s 200 6 s segments in any of the frequency bands, 19 leads were paired as two by two, and a 19 × 19 PLV*_*EEG*_* functional connectivity matrix was calculated for each segment’s EEG, excluding duplicate PLV*_*EEG*_* features that made comparisons with the leads themselves, to obtain a total of 171 PLV*_*EEG*_* features for the EEG recordings of a given subject. The PLV*_*EEG*_* features from 80 segments were then averaged. A total of 707 clinical-PLV*_*EEG*_* features, including 684 PLV*_*EEG*_* and 23 clinical features, were obtained in the four frequency bands for each subject. However, it was unknown which features were valid for a particular learning algorithm, and for this reason, we needed to filter all the features to select those that were beneficial to the learning algorithm. Filtering features not only optimized the algorithm to make the model more generalized but also reduced the running time of the algorithm resolving overfitting issues and the difficulty of the learning task, thereby improving the efficiency and the interpretability of the model.

Fisher score is a common feature filtering method (Zhang J. et al., 2021). Features with a strong discriminatory performance exhibit the smallest possible intra-class distance and the largest possible inter-class distance. The higher the inter-class variance and the lower the intra-class variance of PLV*_*EEG*_* features in the same frequency band from different patients, the higher the Fisher score value is. We ranked the features from the largest to the smallest, based on their Fisher score values, with the higher ranked features being theoretically more discriminative.

### Modeling process

The classification models were trained using AdaBoost and GBDT platforms as classifiers. Models were constructed based on the pure clinical features, PLV*_*EEG*_* features, and combined clinical- PLV*_*EEG*_* features, as well. To improve the classification performance, generalization skills, and speed of each model, Fisher scores were used to filter the features. Five-fold cross-validation was used to construct the classification model, using 80% of the two sets of data each time, and the remaining 20% of the data was used for model validation.

### Statistical analysis

To compare the variability of clinical and normalized PLV*_*EEG*_* features between the CON and CI groups, the quantitative data were first tested for normality using the Shapiro–Wilk test, followed by a comparison of the data with a normal distribution expressed as mean ± standard deviation (SD) using the independent samples *t*-test, and the Mann–Whitney *U*-test was applied for data with an abnormal distribution expressed as median ± interquartile range (IQR). For qualitative information, the chi-squared (χ^2^) test or Fisher’s exact test was used to assess the variability between the two data sets. A *p*- or *p’*- value of < 0.05 was considered statistically significant, where *p’* referred to a *p*-value that was corrected by the false discovery rate (FDR) correction. We used SPSS v26.0 for all kinds of statistical analyses.

## Results

### Clinical feature-based model construction

**FIGURE 3:**
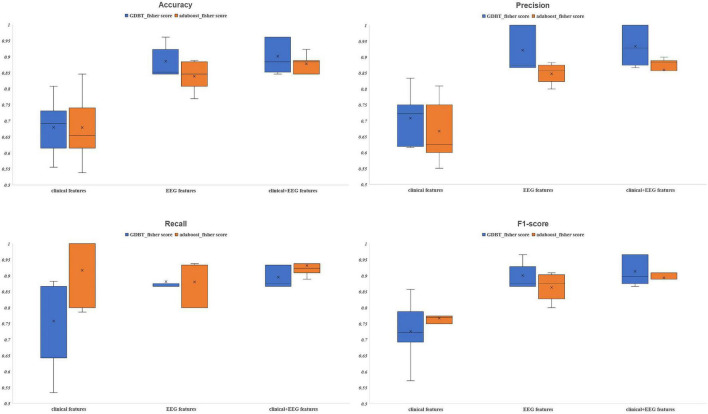
The evaluation indexes after five-fold cross-validation. GBDT, Gradient Boosting Decision Tree; AdaBoost, Adaptive Boosting.

**FIGURE 4:**
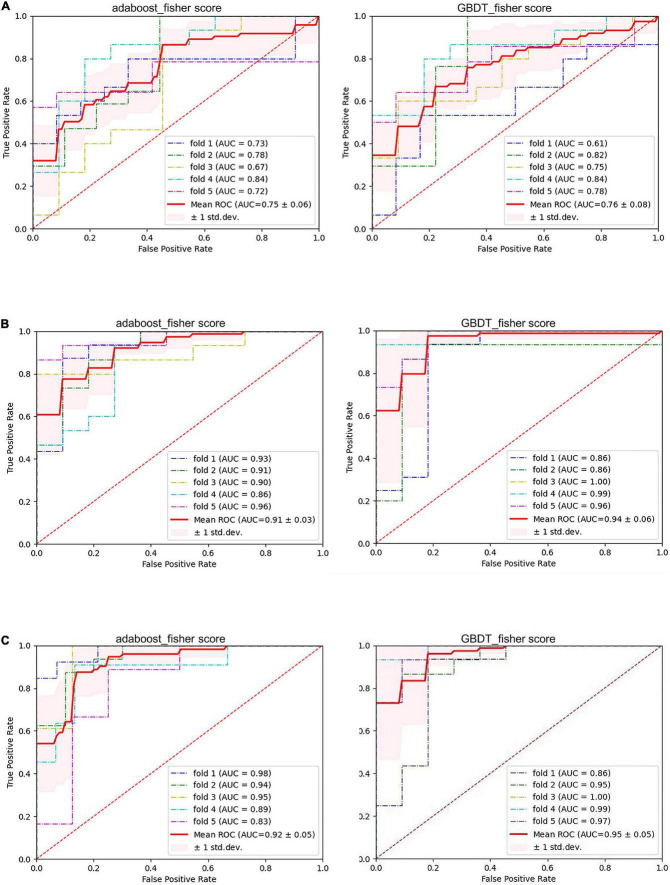
The performance of six models. **(A)** Pure clinical features. **(B)** Pure electroencephalogram (EEG) features. **(C)** Combined clinical and PLV*_*EEG*_* features. GBDT, Gradient Boosting Decision Tree; AdaBoost, Adaptive Boosting; AUC, area under the curve; ROC, receiver operating-characteristic curve; std. dev, standard deviation.

Of the 23 clinical features, we used Fisher scores to filter the top 15 clinical features in terms of weightage to construct the diagnostic model (Table 4A). The selected features were educational attainment, seizure frequency, VPA, class of antiseizures medications, hippocampal atrophy and sclerosis, age, status epilepticus, MRI of the head for abnormalities, time from the first onset to the current visit, history of previous CNS infections, TLE, anxiety, age at the first onset, history of previous head surgeries or trauma, and gender. The features that showed significant statistical differences between the two groups were educational attainment, seizure frequency, VPA, class of antiseizures medications, hippocampal atrophy and sclerosis, and time from the first onset to the current visit. In the classification model, constructed based on the clinical features using AdaBoost, the model performances after a five-fold cross-validation for accuracy, precision, recall, F1-score, and AUC were 67.89, 66.69, 91.57, 76.71, and 0.75%, respectively. While, in case of the classification model built by GBDT, the final performances after cross-validation for accuracy, precision, recall, F1-score, and AUC were, respectively, 68.09, 70.80, 75.84, 72.62, and 0.76% (Figure 3 and Figure 4A). Therefore, these two algorithms were found to differ slightly in the construction of a model for identifying impaired consciousness in epilepsy patients using the clinical features only.

**TABLE 4:** Ranking table of features affecting the model performance.

Rank	Clinic feature	FS-value	Rank	Clinic feature	FS-value
**(A) Top 15 features affecting the pure clinical feature-based model.**					
1	Educational attainment	0.2092	9	Time from first onset to current visit	0.0257
2	Seizure frequency	0.1037	10	History of previous CNS infections	0.0254
3	VPA	0.1033	11	TLE	0.0172
4	Class of antiepileptic drugs	0.0673	12	Anxiety	0.0134
5	Hippocampal atrophy, sclerosis	0.0558	13	Age at first onset	0.0128
6	Age	0.0453	14	History of previous head surgery or trauma	0.0118
7	Status epilepticus	0.038	15	Gender	0.0108
8	MRI of the head for abnormalities	0.0268			
**Rank**	**EEG feature**	**FS-value**	**Rank**	**EEG feature**	**FS-value**
**(B) Top 20 features affecting pure PLV*_*EEG*_*- based feature model.**					
1	θ_T5-T6	0.1191	11	θ_F4-F7	0.0816
2	θ_Fp1-Pz	0.1082	12	θ_Fp2-T6	0.0815
3	δ_Fp1-Pz	0.1076	13	δ_F4-F7	0.0793
4	β_P3-F4	0.1003	14	α_Fp2-T4	0.079
5	β_C3-F4	0.0911	15	θ_P3-F8	0.079
6	α_Fp1-F8	0.0907	16	β_Fp1-F8	0.0787
7	β_F4-F7	0.0848	17	θ_P3-F4	0.0785
8	α_P3-T4	0.0829	18	θ_Fp1-F8	0.078
9	θ_P3-C4	0.0826	19	α_O2-C3	0.0764
10	α_Fp1-F7	0.082	20	β_Fp1-F3	0.0737
**Rank**	**Features**	**FS-value**	**Mean ± STD**	***P*-value**	***P’*-value**
**(C) Features affecting the top 5% of the clinical-PLV*_*EEG*_* feature-based model.**					
1	MRI of the head for abnormalities	0.211	0.557 ± 0.497	0.061	<0.001*
2	Educational attainment	0.194	2.748 ± 1.108	<0.001	0.004*
3	β_C3-F4	0.077	0.155 ± 0.058	0.154	0.265
4	Seizure frequency	0.072	1.359 ± 0.820	0.006	0.052
5	θ_Fp1-Fz	0.072	0.205 ± 0.195	<0.001	<0.001*
6	Hippocampal atrophy, sclerosis	0.069	0.382 ± 0.486	0.004	0.019*
7	β_F3-F8	0.067	0.146 ± 0.048	0.216	0.411
8	β_C3-P4	0.059	0.205 ± 0.074	0.160	0.074
9	θ_C3-P4	0.057	0.237 ± 0.067	0.345	0.156
10	β_T5-T6	0.056	0.139 ± 0.049	0.028	0.012*
11	θ_P4-T5	0.054	0.220 ± 0.116	0.045	0.038*
12	θ_Fp2-T6	0.053	0.235 ± 0.099	0.003	0.008*
13	β_T5-F7	0.052	0.151 ± 0.074	0.028	0.019*
14	β_P3-P4	0.050	0.132 ± 0.050	0.830	0.655
15	VPA	0.049	0.435 ± 0.496	0.013	0.369
16	β_F4-F7	0.048	0.159 ± 0.067	0.179	0.220
17	β_O1-T6	0.047	0.146 ± 0.064	0.282	0.106
18	Class of antiepileptic drugs	0.046	0.450 ± 0.498	0.016	0.125
19	θ_F3-F8	0.046	0.211 ± 0.057	0.467	0.213
20	θ_F4-F7	0.044	0.228 ± 0.067	<0.001	<0.001*
21	δ_P4-T5	0.043	0.293 ± 0.068	0.172	0.321
22	δ_F4-F7	0.042	0.305 ± 0.070	0.009	0.015*
23	β_Fp1-F8	0.042	0.282 ± 0.128	0.579	0.352
24	Time from first onset to current visit	0.040	9.290 ± 8.087	0.009	<0.001*
25	β_P3-F4	0.039	0.315 ± 0.146	0.006	<0.001*
26	Age	0.038	29.260 ± 12.746	0.061	0.075
27	θ_P3-F4	0.038	0.347 ± 0.169	0.013	0.049*
28	β_Fp2-T6	0.038	0.172 ± 0.065	0.130	0.063
29	θ_T5-F7	0.037	0.230 ± 0.082	0.012	0.025*
30	θ_Fp1-T6	0.036	0.290 ± 0.200	<0.001	<0.001*
31	δ_Fp2-T6	0.035	0.320 ± 0.085	0.450	0.157
32	β_Fp1-C3	0.035	0.144 ± 0.050	0.784	0.842
33	θ_O2-Pz	0.034	0.282 ± 0.095	0.211	0.082
34	α_C3-P4	0.034	0.260 ± 0.039	0.331	0.312
35	β_Fp2-F4	0.033	0.359 ± 0.096	0.093	0.165
36	θ_Fp1-F8	0.033	0.332 ± 0.121	0.046	0.025*

FS-value, Fisher score value; α, alpha; β, beta; δ, delta;θ, theta; For qualitative data, Chi-square tests were used; For normal data independent sample *t*-tests were used.

δ Fp1-Fz: δ band from Fp1-Fz and so on; *p* and *p*’ < 0.05 is considered statistically significant, *p*’ refers to *p*-value that is corrected by false discovery rate (FDR) correction. Although the selected features may not be statistically significant, they did have a classification value in the model.

*Is defined as features that have statistically significant between CI group and CON group.

### PLV*_*EEG*_* feature-based model construction

A total of 171 PLV*_*EEG*_* features were extracted for each of the 4 bands of the 20-min EEG recording for each patient, accounting for a total of 684 features (Table 4B). Then the model was constructed using those features with Fisher scores in the top 150 ranks. In the AdaBoost-based classification model, the model performance after a five-fold cross-validation for accuracy, precision, recall, F1-score, and AUC were 83.93, 84.76, 88.08, 86.30, and 0.91%, respectively. Likewise, for the GBDT-based classification model, the final performances after the cross-validation for accuracy, precision, recall, F1-score, and AUC were 88.58, 92.17, 88.17, 90.05, and 0.94%, respectively (Figure 3 and Figure 4B). Importantly, the GBDT was found to outperform AdaBoost in classification model construction using PLV*_*EEG*_* features, demonstrating that the GBDT-based model could be more accurate in identifying epilepsy patients suffering from cognitive dysfunctions. It was also found that PLV*_*EEG*_* features in θ band T5-T6, θ band Fp1-Pz, δ band Fp1-Pz, β band P3-F4, and β band C3-F4 were the top 5 most important ones that might influence the model.

### A combined clinical-PLV*_*EEG*_* feature-based model construction

**FIGURE 5:**
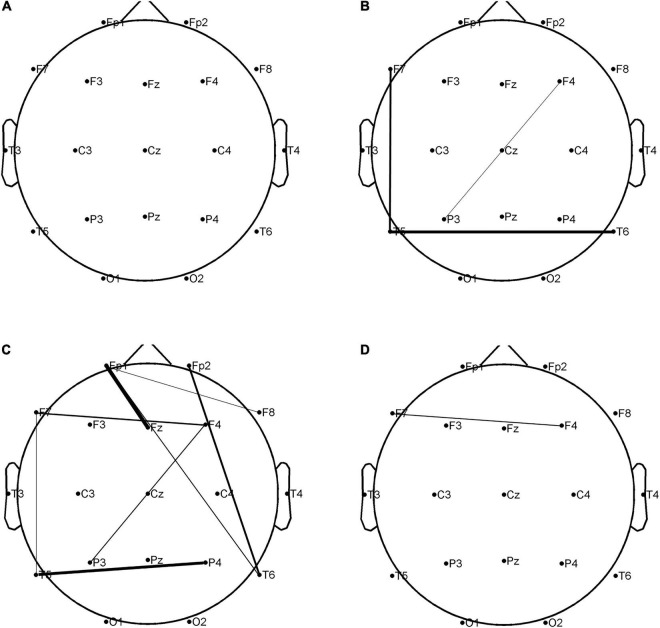
In the combined clinical-PLV*_*EEG*_* model, there were statistically significant differences in 12 PLV*_*EEG*_* features between the CON and CI groups of PWEs. The higher the fisher score, the tighter the connection between the leads. *P* < 0.05 is considered statistically significant. **(A)** Alpha band; **(B)** beta band; **(C)** theta band; **(D)** delta band. PWEs, patients with epilepsy; PLV, phase locking value; CI, cognitive impairment; CON, cognitively normal.

The combined clinical-PLV*_*EEG*_* features were found the most appropriate for constructing the best performing classification models, using either AdaBoost or GBDT algorithm. A total of 707 features were screened using Fisher scores for 23 clinical features and 684 PLV*_*EEG*_* features. A total of 4 clinical features were selected within the top 10 weighted features, namely MRI of the head for abnormalities in the first rank, educational attainment in the second rank, seizure frequency in the fourth rank, and hippocampal atrophy or sclerosis in the sixth rank; all of which were significantly differed between the two groups. Between the two groups, the remaining PLV*_*EEG*_* features with significant differences were C3-F4 in the β-band, Fp1-Pz in the θ-band, F3-F8 in the β-band, C3-P4 in the β-band, C3-P4 in the θ-band, and T5-T6 in the β-band, with only Fp1-Pz in the θ-band, and T5-T6 in the β-band. Although many features were not statistically different between the two groups, they exhibited a very strong impact on the model after the Fisher score screening. Whereas a total of 12 PLV*_*EEG*_* features in the top 5% of features affecting the model performance were significantly different between the two groups, including eight features in the θ band and three PLV*_*EEG*_* features in the β band. We suspected that PLV*_*EEG*_* in the θ band might be the biomarker that could distinguish between these two groups (Table 4C and Figure 5).

For AdaBoost, the top 150 Fisher scores were selected to build the classification model, and the final performances after five-fold cross-validation were 87.78, 85.95, 93.17, 89.35, and 0.92% for accuracy, precision, recall, F1-score, and AUC, respectively. While for GBDT, the top 250 Fisher scores were selected to build the classification model, and the model performances after five-fold cross-validation were 90.11, 93.40, 89.50, 91.39, and 0.95% for accuracy, precision, recall, F1-score, and AUC, respectively (Figure 3 and Figure 4C). The recall performance of the AdaBoost model was found to be slightly higher than that of the GDBT, while GDBT outperformed AdaBoost in terms of other metrics.

### Comparison between different models

Six models, based on the clinical features only, PLV*_*EEG*_* features only, and combined clinical-PLV*_*EEG*_* features, were constructed for 55 CON and 76 epilepsy patients suffering from cognitive dysfunctions, using the ensemble algorithms like AdaBoost and GBDT. We found that the models constructed with combined clinical-PLV*_*EEG*_* features outperformed those developed with either pure clinical or pure PLV*_*EEG*_* features for both the AdaBoost and GBDT algorithms. Notably, the models constructed solely with clinical features performed the worst. The cross-sectional comparisons also revealed that GBDT-built models outperformed the AdaBoost-based ones in both classification models constructed with PLV*_*EEG*_* features. Furthermore, GBDT also outperformed AdaBoost in cases of both pure clinical features and combined clinical-PLV*_*EEG*_* features, with an exception for recall performance (Table 5).

**TABLE 5:** The performance of the six classifier models.

Features and algorithms	Performance	Fold 1	Fold 2	Fold 3	Fold 4	Fold 5	Mean-value
Clinical features GBDT	Accuracy (%)	55.56	80.77	61.54	73.08	69.23	68.03
	Precision (%)	61.54	83.33	61.90	72.22	75.00	70.80
	Recall (%)	53.37	88.23	86.67	86.67	64.28	75.84
	F1-score (%)	57.17	85.71	72.22	78.79	69.23	72.62
	AUC	0.61	0.82	0.75	0.84	0.78	0.76
Clinical features AdaBoost	Accuracy (%)	74.07	84.62	65.38	61.54	53.85	67.89
	Precision (%)	75.00	80.95	62.50	60.00	55.00	66.69
	Recall (%)	80.00	100.00	100.00	100.00	78.57	91.57
	F1-score (%)	77.42	89.47	76.92	75.00	64.71	76.71
	AUC	0.73	0.78	0.67	0.84	0.72	0.75
EEG features GBDT	Accuracy (%)	85.19	84.62	92.31	96.15	84.62	88.58
	Precision (%)	87.50	86.67	100.00	100.00	86.67	92.17
	Recall (%)	87.50	86.67	86.67	93.33	86.67	88.17
	F1-score (%)	87.50	86.67	92.86	96.55	86.67	90.05
	AUC	0.86	0.86	1.00	0.99	0.96	0.94
EEG features AdaBoost	Accuracy (%)	88.89	84.62	80.77	76.92	88.46	83.93
	Precision (%)	88.24	82.35	85.71	80.00	87.50	84.76
	Recall (%)	93.75	93.33	80.00	80.00	93.33	88.08
	F1-score (%)	90.91	87.50	82.76	80.00	90.32	86.30
	AUC	0.93	0.91	0.90	0.86	0.96	0.91
Clinical+EEG features GBDT	Accuracy (%)	85.19	84.62	96.15	96.15	88.46	90.11
	Precision (%)	87.50	86.67	100.00	100.00	92.86	93.40
	Recall (%)	87.50	86.67	93.33	93.33	86.67	89.50
	F1-score (%)	87.50	86.67	96.55	96.55	89.66	91.39
	AUC	0.86	0.95	1.00	0.99	0.97	0.95
Clinical+EEG features AdaBoost	Accuracy (%)	88.89	88.46	92.31	84.62	84.62	87.78
	Precision (%)	85.71	88.24	90.00	76.92	88.89	85.95
	Recall (%)	92.31	93.75	100.00	90.91	88.89	93.17
	F1-score (%)	88.89	90.91	94.74	83.33	88.89	89.35
	AUC	0.98	0.94	0.95	0.89	0.83	0.92

GBDT, Gradient Boosting Decision Tree; AdaBoost, Adaptive Boosting; AUC, area under the curve.

Not only that, but we could also identify potential biomarkers like EEG indicators using the combined clinical-PLV*_*EEG*_* feature-based models that might be able to detect CI in epilepsy patients, which could be highly useful in the diagnosis of epilepsy in clinical settings. Additionally, many of the clinical features used have also been reported in previous studies suggesting their strong association with CI symptoms in epilepsy patients, but have not been ranked to the extent to which these clinical features might affect cognition. Therefore, we ranked these clinical features by their respective Fisher scores. Our findings suggest that EEG could be of great interest to subjects with cognitive deficits, especially those with epileptic symptoms. Previously, technical limitations were the main obstacle in improving the application of EEG for epilepsy diagnosis and treatment. By estimating the combined effects of clinical and PLV*_*EEG*_* features, we could predict the current cognitive status in epilepsy patients, providing clinicians with more options for precise diagnosis and effective treatment plans.

## Discussion

To the best of our knowledge, the present study is the first of its kind to use an integrated algorithm for the construction of a classification model for facilitating the diagnosis of CI in PWE by combined clinical and PLV*_*EEG*_* functional connectivity features.

### Advantages of combined clinical-PLV*_*EEG*_* features for classification model building

Although several risk factors affecting cognitive functions in epilepsy have been identified, however, only a few studies have used these clinical features to predict whether PWEs have a comorbid CI situation. Importantly, it’s been difficult to determine the extent to which these clinical features might affect cognition with a background of epilepsy. A meta-analysis (Novak et al., 2022a) has found that duration of epilepsy, frequency of seizures, and use of antiseizures medications are important clinical features that can affect cognition. Moreover, some studies suggest that education, history of surgical head trauma, anxiety and depression, hippocampal abnormalities, TLE, and seizure types may influence cognitive functions in PWEs (Piazzini et al., 2006; Bell et al., 2011; Vrinda et al., 2019; Jarcuskova et al., 2020; Wang et al., 2020; Phuong et al., 2021; Elsherif and Esmael, 2022). A previous study (Lin et al., 2021) collected 12 clinical features from outpatients with epilepsy to construct a model for diagnosing CI with a performance accuracy, recall, precision, and AUC of 60, 51, 88, and 0.71%, respectively, and concluded that status epilepticus, history of previous surgical head trauma, and seizure frequency were the top three clinical features affecting cognition. However, the clinical features considered in this study were not comprehensive enough, for example, it did not take into account important factors affecting PWEs such as education level and the classes of antiseizures medications taken (Wang et al., 2020). It was previously thought that VPA, PHT, and TPM could cause cognitive dysfunctions in PWEs (Brunbech and Sabers, 2002; Dang et al., 2021; Lozano-Garcia et al., 2021), and for this reason, the presence or absence of these three drugs was used as a clinical feature. The study showed that only VPA had significant weightage for this model, while PHT and TPM, probably due to insufficient data, were not statistically significant, and did not contribute to the construction of the model.

Of the models constructed using pure clinical features, the performance accuracy, recall, precision, and AUC for the AdaBoost/GBDT models were 67.89/68.03%, 91.57/75.84%, 66.69/70.80%, and 0.75/0.76%, respectively. Using Fisher scores, we selected 23 clinical features. Of these, education level, seizure frequency, and VPA therapy ranked the top three clinical characteristics affecting cognition in PWEs. Among the models constructed with combined clinical and PLV*_*EEG*_* features, the accuracy, recall, precision, and AUC of the AdaBoost/GBDT models were 87.78/90.11%, 93.17/89.50%, 85.95/93.40%, and 0.92/0.95%, respectively. We applied the Fisher scoring method for the 23 clinical features and 684 PLV*_*EEG*_* features to jointly screen and rank. Among these features, MRI abnormalities, education level, and seizure frequency were the top 3 most influential clinical features. The performance of the models constructed using clinical features alone was better than that shown in previous studies for all metrics, except for the performance accuracy. While the performance of the models constructed using combined clinical and PLV*_*EEG*_* features was significantly improved than that reported previously. Thus, we concluded that combined clinical and PLV*_*EEG*_* features were more appropriate for PWEs and that a combination of different types of features would be an optimal choice for constructing diagnostic prediction models.

### PLV*_*EEG*_* features are valid indicators for diagnosing CI in PWEs

PLV*_*EEG*_* is used to remotely examine the task-induced changes in neural activities, synchronized in EEG recordings, which is a classic metric for computing functional brain connectivity features. Jones et al. (2022) have used PLV*_*EEG*_* functional connectivity features as an evaluation metric for assessing the efficacy of transcranial alternating current stimulation (tACS) on age-associated cognitive decline. Li et al. (2022) have constructed a model combining the clinical and PLV*_*EEG*_* features to diagnose Alzheimer’s disease (AD), which exhibits satisfactory performance and robustness. Another study (Lanzone et al., 2021) has found that PLV*_*EEG*_* in the α band of patients who were effective on treatment with perampanel as an add-on drug could be used as a biomarker to predict the responsiveness to perampanel drugs. Cho et al. (2017) have reported that PLV*_*EEG*_* in the γ band may be a potential biomarker for predicting seizures. In this study, the accuracy, recall, precision, and AUC of the AdaBoost/GBDT models were 83.93/88.58%, 84.76/92.17%, 88.08/88.17%, 86.30/90.05%, and 0.91/0.94%, respectively, when only the PLV*_*EEG*_* features were used for the model construction. The θ-band T5- T6, θ-band Fp1-Pz, and δ-band Fp1-Pz were the top three PLV*_*EEG*_* features affecting the model weightage, indicating that the PLV*_*EEG*_* functional connectivity features might be valid indicators for the diagnosis of cognitive dysfunctions comorbid with epilepsy.

### PLV*_*EEG*_* features in the θ band may be a potential biomarker for diagnosing CI in PWEs

Here, we calculated the PLV*_*EEG*_* features of the four frequency bands (α, β, θ, δ), and found that the PLV*_*EEG*_* features, especially of the θ band, might be potential biomarkers to distinguish between epilepsy patients with or without comorbid CI. In our constructed model of the combined clinical and PLV*_*EEG*_* features, we employed Fisher scoring to rank individual features, which revealed 12 PLV features that ranked in the top 30 were significantly different between the CON and CI groups. Notably, eight of these features were related to the θ band and three to the β band.

The θ band has been found to have an important relationship with epilepsy and cognitive function in previous studies. One study (Douw et al., 2010) has demonstrated that functional connectivity features in the θ band could be used to aid in the diagnosis of epilepsy with a recall of 62% and a specificity of 72%. Other studies (Jun et al., 2020) have also suggested that stimulation of the hippocampus may increase the release of θ rhythms, thereby improving the associative memory function. These studies suggest that increasing the θ rhythm in the hippocampus may provide a theoretical basis for the neural mechanisms of memory enhancement. Moreover, Gupta et al. (2012) have identified that θ rhythms in the hippocampus of rats are associated with visuospatial abilities and executive abilities related to memory and cognition. Another study (Braithwaite et al., 2020) has revealed that increased power of the θ rhythm in children can be a valid biomarker for predicting non-verbal cognitive abilities. Furthermore, it (Ahmadlou et al., 2014) has been concluded that functional connectivity features in the θ band could be used to differentiate between patients with mild CI and healthy elderly populations. Briels et al. (2020) have found that functional connectivity indicators in the θ and β frequency bands in AD patients may help diagnose the disease severity. Other studies (Singh et al., 2018) have shown that a reduction in midfrontal θ wave frequency responds to the degree of effective control of cognitive functions in PD patients. The θ rhythms in the frontal lobe are highly correlated with cognitive function (Cavanagh and Frank, 2014), with Fp1-Fz being within the frontal lobe. Our results showed that the PLV*_*EEG*_* features of Fp1-Fz in the θ band were significantly different between the CON and CI groups of epilepsy patients, accounting for a high weightage in the diagnostic model. In this context, one study (Cao et al., 2022) has reported an important relationship between the θ rhythm and cognition in patients with schizophrenia, indicating that superior cognitive performance may be significantly associated with a smaller θ wave power, and altered θ rhythm and cognition are highly correlated mainly in the parieto-occipital lobe. The P4 and T5 were close to the occipital region in our investigation. The PLV*_*EEG*_* for P4-T5 were also significantly different between the two groups and accounted for a higher weightage in the model. Furthermore, it is shown (Usami et al., 2019) that β oscillations can enhance the responsiveness of the cerebral cortex to inputs from distant cortices, suggesting that β frequencies may have an important role in functional connectivity. Interestingly, α frequency is significantly increased in AD patients presenting with mild cognitive dysfunctions (Moretti, 2015). The α frequency was found to be less influential in our study, in terms of statistical significance and the weightage of the model, possibly due to the exclusion of AD patients’ data.

Previous studies have amply demonstrated the significance of functional connectivity features in the θ band in the diagnosis of epilepsy and cognitive dysfunctions. Therefore, our study demonstrated that PLV*_*EEG*_* features in the θ band might be reliable biomarkers for diagnosing CI in PWEs, especially those with high Fisher scores.

### Limitations and future directions

Despite these excellent results, there are still certain limitations to this study. First, this was a single-center retrospective study with data from only one institutional epilepsy center and a small sample population. Although the combined clinical and PLV*_*EEG*_* features and advanced algorithms ensured the accuracy of our results, multi-center prospective studies are warranted for the generalization of our results. Here, we provided a theoretical basis and demonstrated the possibilities of further improving the diagnostic methods for PWEs comorbid with CI. Second, this study was based on the MoCA scale. However, we classified the features based on the total MoCA scores rather than the subtest scores. Although our model could address the issue of differentiating PWEs with or without cognitive deficits, the content of each subtest should be investigated more carefully in the future. Finally, the potential biomarkers that we extracted were mainly functional connectivity features of the EEG and a subset of clinical features. The future brain network features extracted from MRI examinations can be useful in improving the accuracy and superiority of the model. We propose to validate the performance of our models with larger datasets from multiple epilepsy centers in the future, as well as add new features to improve the accuracy of the model.

## Conclusion

In this study, we constructed a diagnostic model for CI in PWEs based on the combined clinical and PLV*_*EEG*_* features. Besides, we found that PLV*_*EEG*_* functional connectivity features in the θ band might be potential biomarkers for the diagnosis of CI in PWEs.

## Data availability statement

The raw data supporting the conclusions of this article will be made available by the authors, without undue reservation.

## Ethics statement

The studies involving human participants were reviewed and approved by The Institutional Review Board of the Henan Provincial People’s Hospital. Written informed consent to participate in this study was provided by the participants’ legal guardian/next of kin.

## Author contributions

ZR and MY designed the study. XH obtained funding. ZR, YZ, YH, YXH, and QW acquired the data. PZ, TZ, and NW analyzed EEG recordings. ZR, ZZ, and BWe worked on EEG preprocessing and machine learning process. ZR, ZZ, and BWa conducted the statistical analysis. ZR, ZZ, YH, YXH, and QW analyzed and interpreted the data. ZR and XH drafted and revised the manuscript. All authors revised this draft and read and approved the final manuscript.
